# Co-occurrence of pathogen assemblages in a keystone species the common cockle *Cerastoderma edule* on the Irish coast

**DOI:** 10.1017/S0031182021001396

**Published:** 2021-11

**Authors:** Sara Albuixech-Martí, Sarah C. Culloty, Sharon A. Lynch

**Affiliations:** 1 School of Biological, Earth & Environmental Sciences, University College Cork, Cooperage Building, Distillery Fields, North Mall, Cork, Ireland; 2 Aquaculture & Fisheries Development Centre, University College Cork, Cooperage Building, Distillery Fields, North Mall, Cork, Ireland; 3 Centre for Marine and Renewable Energy Ireland (MaREI), Beaufort Building, Environmental Research Institute (ERI), University College Cork, Ringaskiddy, Cork, Ireland.

**Keywords:** Cockle health, coinfection, confounding factors, Haplosporidia, pathogen interactions, *Vibrio*

## Abstract

Despite coinfections being recognized as the rule in animal populations, most studies focus on single pathogen systems. Pathogen interaction networks and the drivers of such associations are lacking in disease ecology studies. Common cockle *Cerastoderma edule* populations are exposed to a great diversity of pathogens, thus making them a good model system to investigate. This study examined the diversity and prevalence of pathogens from different taxonomic levels in wild and fished *C. edule* on the Irish coast. Potential interactions were tested focussing on abiotic (seawater temperature and salinity) and biotic (cockle size and age, and epiflora on shells) factors. No Microsporidia nor OsHV-1*μ*Var were detected. Single infections with Haplosporidia (37.7%) or *Vibrio* (25.3%) were more common than two-pathogen coinfected individuals (9.5%), which may more easily succumb to infection. Fished *C. edule* populations with high cockle densities were more exposed to infections. Higher temperature and presence of epiflora on cockle shells promoted coinfection in warmer months. Low seawater salinity, host condition and proximity to other infected host species influenced coinfection distribution. A positive association between two *Minchinia* spp. was observed, most likely due to their different pathogenic effect. Findings highlight the major influence that ecological factors have on pathogen interactions and host–pathogen interplay.

## Graphical abstract

**Figure:**
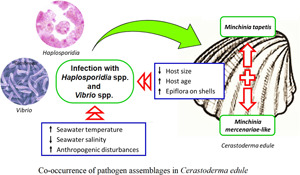

## Introduction

Animal hosts are exposed to complex pathogen assemblages that ultimately form a dynamic community within them (Johnson and Buller, 2011). However, research into host–pathogen interactions remains dominated by the study of one host–one pathogen systems despite the fact that hosts can simultaneously carry several infectious agents, with consequences for the dynamics of each agent and host health. Coinfections have been previously reported in shellfish hosts: including *Vibrio tapetis*, *Perkinsus* sp. and digenetic trematodes in cockles (Lassalle *et al*., 2007); ostreid herpesvirus-1 (OsHV-1) and *Vibrio* spp. in oysters (Petton *et al*., 2015; Alfaro *et al*., 2019); and protozoans and bacteria in clams (Arzul *et al*., 2012; Carella *et al*., 2020); and in some of the cases with a dramatic effect on disease susceptibility (Cox, 2001; Lassalle *et al*., 2007; Arzul *et al*., 2012; Alfaro *et al*., 2019). Processes at different scales of ecological organization from the within-host level (e.g. interactions of coinfecting parasites) to the ecosystem level (e.g. the influence of environmental variables) across space and over time have a key influence in complex natural systems (Hellard *et al*., 2015). Therefore, moving from one host–one pathogen systems towards an ecosystem view of host–pathogen interactions and their ecology is essential.

Detection of interspecific parasite/pathogen interactions in natural populations is not easy, due to complex networks of indirect effects making it difficult to infer underlying processes (Hellard *et al*., 2012). Interactions among coinfecting infectious agents can, in fact, alter host pathology, parasite transmission and virulence evolution, also influencing the spread of infections at a population level resulting in disease outbreaks, as described previously in bivalves (Lassalle *et al*., 2007; Arzul *et al*., 2012; Alfaro *et al*., 2019). These effects can be the opposite, i.e. coinfections within the host may reduce infection success yet still enhance pathology or, in contrast, co-occurring parasites/pathogens may weaken host pathology increasing the infection success (Johnson and Hoverman, 2012). For example, mortality induced by one parasite can also eliminate the availability of hosts for other parasites (Jolles *et al*., 2008); likewise, morbidity induced by one parasite can increase exposure to a second, even if within-host interactions are antagonistic (Karvonen *et al*., 2009).

Simultaneous infections may therefore trigger a whole spectrum of outcomes within the hosts, both synergistic and antagonistic, i.e. the weight of one or both infectious agents may be suppressed (Fukami *et al*., 2010), one or both agents may be amplified resulting in coexistence (Thomas *et al*., 1998), or one may be amplified and the other suppressed (Johnson and Hoverman, 2012). Parasite/pathogen interactions can be not only direct, such as competition for attachment sites, competition for host resources and predation upon one another (intra-guild predation) (Hatcher *et al*., 2006, 2012; Johnson *et al*., 2010), but interactions can also be indirect or host-mediated often involving changes in immunity such as cross-immunity (apparent competition) and immune suppression (apparent facilitation) (Jolles *et al*., 2008). For instance, Johnson and Hoverman (2012) suggested cross-reactive immunity as a cause of a decrease in parasite persistence in their experiments with a group of related larval trematodes in Pacific chorus frogs (*Pseudacris regilla*). Johansen and Sommer (2001) suggested that the immune suppression triggered by a primary infection could be the cause of multiple bacterial infections in Atlantic salmon (*Salmo salar*).

The co-occurrence of infectious agents may also result simply because the same risk factors promote their presence and not because the different pathogen groups are interacting synergistically (Vaumourin *et al*., 2015). Risk factors shared by two or more infectious agents can occur at the level of the host individual (e.g. host condition and age), population (e.g. host density) or landscape (e.g. climatic factors), increasing the probability of infection by both/all agents (Hellard *et al*., 2015). Such confounding factors may influence host exposure and/or host susceptibility promoting simultaneous infections (Vaumourin *et al*., 2015). Environmental parameters such as seawater temperature and salinity, host density or host physiological conditions and behaviour are common confounding factors that may influence the co-occurrence of different pathogen groups and the epidemiological and geographic patterns of infection and disease (Hellard *et al*., 2012; Vaumourin *et al*., 2015). Confounding factors must be considered in field studies since those factors can create statistical associations between infectious agents even if there is no true biological interaction between them (Kuris and Lafferty, 1994).

Generalist host species, such as the common cockle *Cerastoderma edule*, with wide spatial distributions, are exposed to a large range of environmental conditions, and a greater diversity of parasites and pathogens, and consequently are more likely to be coinfected (Vaumourin *et al*., 2015; Mahony *et al*., 2020; de Montaudouin *et al*., 2021), directly affecting individual cockle health and cockle population dynamics (Longshaw and Malham, 2013). Cockles may be host to multiple macro-parasite infections, particularly trematodes (de Montaudouin *et al*., 2010, 2021), but may also be infected by a range of pathogens (Rowley *et al*., 2014; de Montaudouin *et al*., 2021), providing a microcosm for the parasite/pathogen community. Within this community, there are haplosporidian protists such as the genus *Minchinia* (Engelsma *et al*., 2011; Longshaw and Malham, 2013; Ramilo *et al*., 2018; Lynch *et al.*, 2020*b*; Albuixech-Martí *et al*., 2020; de Montaudouin *et al*., 2021), numerous members of the bacterial family *Vibrio* (Carballal *et al*., 2001; Lassalle *et al*., 2007; de Montaudouin *et al*., 2021), viruses such as ostreid herpesvirus-1 microVar (OsHV-1 *μ*Var) (Carballal *et al*., 2003; Evans *et al*., 2017; Bookelaar *et al*., 2020; de Montaudouin *et al*., 2021) and microsporidian species reported in digeneans and paramyxids infecting cockle populations (Goater, 1993; Fermer *et al*., 2009, 2011; Villalba *et al*., 2014; de Montaudouin *et al*., 2021). Cockles are a keystone species responsible for different ecosystem services, i.e. carbon storage, energy cycling, food source for seabirds, etc., being also good sentinel and bioindicator species (Malham *et al*., 2012; Carss *et al*., 2020). Moreover, the common cockle is one of the main non-cultured bivalve species harvested in western European waters, with a high economic value (Rowley *et al*., 2014; Carss *et al*., 2020). Consequently, cockles have been well-studied and are useful model species. Given the complex parasite/pathogen assemblages within the cockles and their intricate interactions and effects, a comprehensive and integrated approach that considers not only the synergistic impact of coinfections but also the role of ecological factors driving the infection across space and over time is needed.

Future climate scenarios predict a warming marine environment and increased precipitation events resulting in pulse events of reduced/fluctuating salinity in nearshore ecosystems (Allam and Raftos, 2015; Coen and Bishop, 2015). Consequently, climate change may be expected to have a significant effect on disease epidemiology (Coen and Bishop, 2015), and may even have significant consequences on zoonoses emergence (Hoarau *et al*., 2020). In this context, the need for the understanding of ecological processes involved in the transmission and dynamics of infectious agents in host reservoirs is critical (Hoarau *et al*., 2020). This study, therefore, aims to assess the interactions between infectious agents, their hosts and the environment.

For that purpose, this study examined the diversity and prevalence of significant pathogen groups, Haplosporidia, *Vibrio* spp., OsHV-1 *μ*Var and Microsporidia, associated with bivalve mortalities globally as well as coinfections and associations of the different infectious agents at an individual and population level in wild and fished *C. edule* along the Irish and Celtic Seas. The role of abiotic (seawater temperature and salinity) and biotic (cockle size and age, and epiflora on their shells) factors in the risk of coinfection throughout the study was also assessed. Developing knowledge on pathogen diversity and co-occurrence in an economically and ecologically important species as is *C. edule* and integrating the role of the environment in the host–pathogen interplay is essential for a better understanding of the interaction networks that can affect disease outcomes and transmissibility in the current changing environment. Findings from this study highlight the complexity of pathogen interactions within a host and the effect of key biotic and environmental drivers shaping disease dynamics, which can also be applied to other coinfected animal host species.

## Materials and methods

### Sampling

**Fig. 1.**
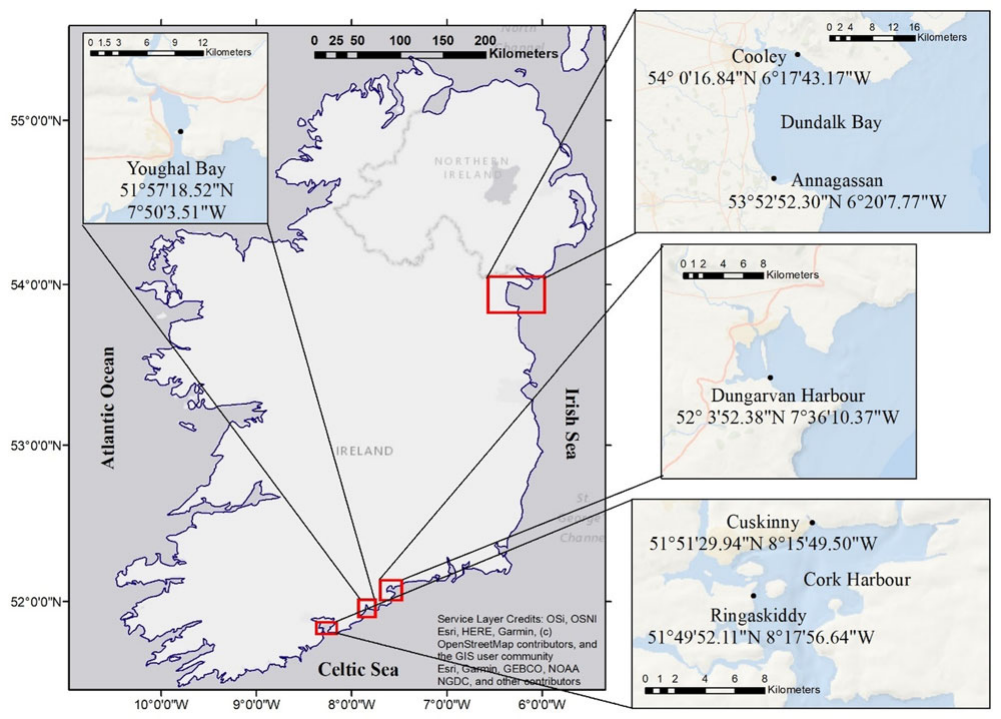
Map of Ireland highlighting the cockle sample sites with coordinates.

Cockles were sampled from spring 2018 to spring 2019 at Cork Harbour (Ringaskiddy and Cuskinny, *n* = 257) on the south coast (Celtic Sea) of Ireland and from summer 2018 to spring 2019 at Youghal Bay (*n* = 69) and Dungarvan Harbour (*n* = 169), with an extensive farming of Pacific oysters (*Crassostrea gigas*), on the south coast (Celtic Sea) of Ireland, where there is no commercial fishing activity (Fig. 1). Additionally, 240 cockles were collected from summer 2018 to spring 2019 at the commercial fishery at Dundalk Bay (Annagassan and Cooley) on the northeast coast (Irish Sea) of Ireland (Fig. 1). Dundalk Bay is a classified Bivalve Mollusc Production Area and it has supported a commercial dredge cockle (*C. edule*) fishery since 2001 (Hervas *et al*., 2008).

The four locations are bays with intertidal sand and mudflats, where a variety of bivalves inhabit. *Cerastoderma edule* densities in the selected sites vary from higher densities, 9.33 ± 3.5 ind m^−2^, in Dundalk Bay (Shellfish Stocks and Fisheries Review, Marine Institute and BIM, 2017) to lower densities in the no commercial fishing sites: 1.6 ± 2.1 ind m^−2^ in Youghal Bay; 0.8 ± 1.7 ind m^−2^ in Cork Harbour; and 0.4 ± 1.3 ind m^−2^ in Dungarvan Harbour (Fermer *et al*., 2011). Cockle mortalities associated with disseminated neoplasia have been previously reported in Cork Harbour (Morgan *et al*., 2012). While a massive cockle mortality event associated with dinoflagellates occurred in Youghal Bay in the past (Ottway *et al*., 1979). Dungarvan Harbour has also a history of OsHV-1-associated mortality events (Lynch *et al*., 2012; Prado-Alvarez *et al*., 2016). However, to the best of our knowledge, Dundalk Bay has not previously recorded mortality events.

Even though Cork Harbour is an important industrial area and is a busy ferry/shipping terminal, its trophic status (based on the nutrient levels such as phosphorus and nitrogen, growth of algae and dissolved oxygen concentration) was qualified as unpolluted by the EPA in 2017 along with Dungarvan Harbour, an extremely sheltered bay, while Youghal Bay, close to an urban hub, was classified as intermediate polluted area. In turn, Dundalk Bay showed a localized effect on the trophic status, with Annagassan classified as an unpolluted area and Cooley as an intermediate polluted area, probably due to the influence of water discharge from the local river.

Cockles were collected by hand raking from the intertidal area of each site at spring tide (0.5–1 m) and seasonal intervals. The sample size, outlined in Table 1, was dependant on the availability of cockles at each site and season. Cockles were kept at ambient temperature during the fieldwork and were processed either on the same day or were held out of water overnight at 4 °C and were processed the next day.

**Table 1.** Sample sites, seasons and number of cockles collected

Sampling dates	Cork Harbour	Youghal Bay	Dungarvan Harbour	Dundalk Bay	Totals
Spring 2018	60	–	–	–	60
Summer 2018	39	11	51	60	161
Autumn 2018	60	16	58	60	194
Winter 2018/19	38	30	30	60	158
Spring 2019	60	12	30	60	162
Totals^a^	257 (34.9%)	69 (9.4%)	169 (23.0%)	240 (32.7%)	735

(–) No samples collected.

a In brackets the % representation of all cockles sampled.

### Sample processing and diagnostic methods

#### Morphometrics

Measurements included (A) cockle body size [height (mm)] of each individual that was recorded using Vernier callipers. The (B) cockle's age was established by counting the annual winter growth rings. For verification and validation purposes, the number of growth rings was confirmed by a second researcher, as it is recognized that variations in growth during the same year lead to the possible formation of extra stria (de Montaudouin, 1996). Ultimately, the presence or absence of epiflora on the shell of each individual was also recorded. Epiflora was mainly composed of epiphytic algae, although no specific species were identified.

#### Histology

A transverse section of each cockle was excised, including the digestive gland, gonads, gills and mantle tissues; as well as a section of the hinge ligament and a section of the tip of the foot were taken for histological examination. The three sections were placed in a labelled histocassette and were preserved in Davidson's fixative for 48 h (Shaw and Battle, 1957) prior to processing. Paraffin-embedded tissue blocks were sectioned at 5 *μ*m and stained using haematoxylin and eosin (Sigma Aldrich, USA) (Howard and Smith, 1983). Tissue slides were screened at a magnification of 40x and under oil at 100x for visualization of the pathogens and tissue pathology.

#### Molecular techniques for cockle species identification and pathological screening

A small piece of gill tissue (2–5 mm^2^ of tissue) from each cockle was taken and stored at −20 °C for molecular assays, to determine the European cockle species [*C. edule* (Linnaeus, 1758) or *Cerastoderma glaucum* (Poiret, 1789)] and for subsequent pathological screening. Genomic DNA from the cockle gill tissue was extracted using the chelex-100 extraction method (Walsh *et al*., 1991; Lynch *et al*., 2008).

Polymerase chain reaction (PCR) was carried out to amplify the nuclear DNA markers *ITS-for*/*ITS Ce-R*/*ITS Cg-R* to differentiate between the presence of *C. edule*, *C. glaucum* species or hybrids (Freire *et al*., 2011; Table 2). Amplification was conducted following the reaction mixture and thermocycling conditions, as well as the visualization of the product, described in Albuixech-Martí *et al*. (2020).

**Table 2.** Description of PCR primer pairs showing sequences for each forward and reverse primer and expected product size

	Primer sequence (5′–3′)			
Primer pair	Forward	Reverse	Product size (bp)	Primer specificity
ITS-for ITS Ce-R	GTT TCC GTA GGT GAA CCT G	AAG CAG CGA GAA GCC GTT C	190	*Cerastoderma edule*
ITS-for ITS Cg-R	GTT TCC GTA GGT GAA CCT G	AAT TCG CCA TCG TCG G	470	*Cerastoderma glaucum*
HAP-F1 HAP-R3	GTT CTT TCW TGA TTC TAT GMA	AKR HRT TCC TWG TTC AAG AYG A	350	Haplosporidia spp.
TAP-For TAP-Rev	ATC TAA CTA GCT GTC GCT AAC TCG T	CTT TCA AGA TTA CCC GGC TCT GC	165	*Minchinia tapetis*
MER-For MER-Rev	ATC TAA CTA GCT GTC ACT ATG GAA AA	ACG CAC ATT AAA GAT TGC CCA GCT CTT T	170	*Minchinia mercenariae*-like
Vib-F3 Vib-R3	CAA CAG AAG AAG CAC CGG CT	CAC GCT TTC GCA TCT GAG TG	286	*Vibrio* spp.
OHVA OHVB	TGC TGG CTG ATG TGA TGG CTT TGG	GGA TAT GGA GCT GCG GCG CT	385	*Ostreid herpesvirus-1 and variants*
MicIF1 MicIR1	GTG GAC GCT AGT CTC ACA GGT T	TTG CAC CAG AAG GTT TAC GAC ACA T	180	*Microsporidia sp.1*
MicIF2 MicIR2	ATG CAT GCG TAA GCG AAG CAG TTA T	TCT CTT GCA CCA GAA GGT TTA CGA C	180	*Microsporidia sp. 2*

Standard PCR screening for haplosporidian detection was carried out in cockle gill tissue samples (*n* = 735) using generic haplosporidian HAP-F1 and HAP-R3 primers that amplify small regions of the *SSU rDNA* of most haplosporidians (Renault *et al*., 2000; Molloy *et al*., 2012; Table 2). Amplification was conducted following the reaction mixture and thermocycling conditions, as well as the visualization of the product, described in Albuixech-Martí *et al*. (2020).

Standard PCR screening for *Minchinia* spp. detection was carried out in the cockle samples that were positive for Haplosporidia by conventional PCR, using specific primers for *Minchinia tapetis* (TAP-For/Rev) and *Minchinia mercenariae*-like (MER-For/Rev) (Albuixech-Martí *et al*., 2020; Table 2) in the appropriate pairings and separate PCR reactions. Amplification was conducted following the reaction mixture and thermocycling conditions, as well as the visualization of the product, described in Albuixech-Martí *et al*. (2020).

Standard PCR screening for *Vibrio* spp. in cockle gill tissues was conducted using generic primers Vib-F3/R3, developed in our lab for the detection of *Vibrio* genus [Kett *et al*., unpublished as per Notaro *et al*. (2021), data confirmed to be specific for *Vibrio* spp. by Sanger sequencing; Table 2]. The screening was conducted in all the collected cockles (*n* = 735). A total of 2 *μ*L of DNA per individual was used in a total volume of 27.5 *μ*L of the reaction mixture containing: 5 *μ*L of 5x green buffer, 5 *μ*L of dNTPs (1.25 mm), 0.5 *μ*L of MgCl_2_, 0.25 *μ*L of forward and reverse primers, 0.1 *μ*L of GoTaq polymerases, 1.5 *μ*L of DMSO and 12.9 *μ*L of ddH_2_O. Cycling conditions consisted of an initial denaturation of the sample at 95 °C for 1 min followed by 35 cycles of 94 °C for 20 s, 56 °C for 30 s, 72 °C for 30 s and a final elongation at 72 °C for 7 min. Electrophoresis of the amplification products was conducted in a 2% agarose gel.

Standard PCR screening for OsHV-1 and variants, including OsHV-1 *μ*Var, was carried out in cockle gill tissue samples (*n* = 735) using specific primers OHVA/OHVB that amplify small products of the *ORF4* gene in the OsHV-1 virus and its variants (Lynch *et al*., 2013; Table 2). Amplification was conducted following the reaction mixture and thermocycling conditions described in Lynch *et al*. (2013). Electrophoresis of the amplification products was conducted in a 2% agarose gel.

Standard PCR using specific primers MicIF1/MicIR1 and MicIF2/MicIR2 (Lynch *et al*., unpublished data confirmed to be specific to Microsporidia spp. by Sanger sequencing; Table 2) to detect two unidentified microsporidian species was carried out in cockle gill tissue samples (*n* = 735). Both reaction mixture and thermocycling conditions were the same as used with the PCR for OsHV-1 and variants, as well as the visualization of the product.

Real-time quantitative PCR (qPCR) for detection and quantification of *Vibrio aestuarianus* in the cockle samples that were positive for *Vibrio* spp. was performed using specific primers (Table 3) for detection of the molecular chaperone *dnaJ* gene, following the protocol of McCleary and Henshilwood (2015). All samples were performed in triplicates using 5 *μ*L of DNA. *C*_t_ values were used to determine real-time PCR quantification and detection limits. A tested sample was considered positive if its mean *C*_t_ value was below 37.

**Table 3.** Description of qPCR primer pairs and probe

Primers	Sequence	Specificity
*dnaJ* f420 *dnaJ* r456 *dnaJ* p441 (probe)	GTGAAGGGACGGGTGCTAAG CCATGACAAGTGCCACAAGTCT FAM-AGGGCACGTCGGC-MGB	*Vibrio aestuarianus*
*HVDP-F* *HVDP-R*	ATTGATGATGTGGATAATCTGTG GGTAAATACCATTGGTCTTGTTCC	*Ostreid herpesvirus-1 and variants*

Viral load for OsHV-1 and variants was investigated by qPCR with a small number of individuals (*n* = 30, whose standard PCR results displayed some smear in the visualization of the product) to verify that there was no viral load by carrying out a more specific screening and increasing the amount of DNA processed. The protocol of Pepin *et al*. (2008) was followed, using the specific primers HVDP-F/HVDP-R (Table 3). qPCR was conducted in duplicate using 5 *μ*L DNA by a Thermo Hybaid PCR express thermal cycler.

Direct sequencing was carried out on representative PCR products (*n* = 10) amplified using generic *Vibrio* Vib-F3/R3 primers (Kett *et al*., unpublished as per Notaro *et al*., 2021) to identify the *Vibrio* species present in the samples. Likewise, direct sequencing was carried out on representative PCR products (*n* = 5) amplified using generic haplosporidian HAP-F1 and HAP-R3 primers (Renault *et al*., 2000; Molloy *et al*., 2012) to identify the species present in the samples beyond the *Minchinia* species screened. Genomic DNA from selected individuals was isolated and purified using the QIAquick Gel Extraction Kit (QIAgen) prior to direct sequencing. Both the forward and reverse strands of DNA samples were sequenced commercially (Source Bioscience). Each sequence was matched against the National Center for Biotechnology Information (NCBI) nucleotide database with BLASTn (Basic Local Alignment Search Tool), which finds regions of local similarity between sequences to identify and confirm the DNA being detected in the PCRs.

### Environmental data

This study was conducted using E.U. Copernicus Marine Service Information (marine.copernicus.eu/services-portfolio/access-to-products). Two environmental parameters critical in shaping host–pathogen interactions in estuarine and marine environments (Coen and Bishop, 2015) were selected for this study as potential confounding factors: seawater temperature and salinity. Recorded data in seawater temperature (°C) and salinity (PSU), taken monthly at a depth of 0.5 m below sea level, were downloaded by ArcGIS Desktop 10.5.1, Redlands, CA, USA (Environmental Systems Research Institute, 2017) throughout the duration of the study (from April 2018 to May 2019) from the different sample sites (coordinate-based) along the Irish coast.

### Statistical analysis

Statistical analysis was performed in R version 3.2.3. statistical software. The pathogen occurrence was modelled as pathogen presence/absence in each cockle for each pathogen group and their combinations, and it was used in the subsequent analysis to examine the associations between the different pathogen groups present through the samples.

Association screening approach (SCN), described in detail by Vaumourin *et al*. (2014), was conducted running 5000 simulations (NS) with the presence/absence of Haplosporidia and *Vibrio* spp. to determine a potential association between these two pathogen groups, under the null hypothesis of random pathogen associations [*P*(SCN) < 0.05]. Subsequently, a second SCN (NS = 5000) was performed with the presence/absence of the different pathogen species detected and quantified in the samples, i.e. *M. tapetis*, *M. mercenariae*-like and *V. aestuarianus*.

Corrected Pearson's *χ*^2^ test, described in detail by Hellard *et al*. (2012), was applied to the Haplosporidia and *Vibrio* spp. presence/absence data to determine if the coinfection was due to an underlying biological interaction or to confounding factors considered (seawater temperature, salinity, cockle size and age, and epiflora on their shells). Likewise, the test was applied to the *M. tapetis* and *M. mercenariae*-like presence/absence data. Biological interactions between two pathogens, therefore, will be suspected when the probability of coinfection is not random once confounding factors have been considered [*P*(Corr *χ*^2^) < 0.05]. In the model, running 1000 bootstraps, the effects of the confounding factors are summarized as F1 + F2 + F3 + F4 + F5.

Moreover, Pearson's *χ*^2^ tests were used to determine whether the prevalence observed of single infections and two-pathogen coinfections was significantly different [*P*(Chisq) < 0.05] among the sample sites and throughout the year. Fisher's exact test was conducted when the frequencies of the pathogen occurrence in a site or season were <4, to gain accuracy.

## Results

In total, 735 cockles were screened to determine the prevalence of infection and coinfections of the target pathogens (Table 4). The cockle speciation PCR confirmed that all the individuals from all sample sites were *C. edule*.

**Table 4.** Prevalence (%, positive individuals/screened individuals) of Haplosporidia spp., *Minchinia tapetis*, *M. mercenariae*-like, *Vibrio* spp. and *Vibrio aestuarianus* screening in *Cerastoderma edule* at each sample site by season

Sample site	Pathogen identification	Spring 2018	Summer 2018	Autumn 2018	Winter 2018/19	Spring 2019	Totals
Cork Harbour – Ringaskiddy and Cuskinny	Haplosporidia spp.	**45.0% (27/60)**^a^	15.4% (6/39)	41.7% (25/60)	21.1% (8/38)	28.3% (17/60)	32.3% (83/257)
	*Minchinia tapetis*	15.0% (9/60)	7.7% (3/39)	**18.3%** (**11/60)**^a^	2.6% (1/38)	1.7% (1/60)	9.7% (25/257)
	*Minchinia mercenariae-*like	**23.3% (14/60)**^a^	0% (0/39)	8.3% (5/60)	0% (0/38)	10.0% (6/60)	9.7% (25/257)
	*Vibrio* spp.^b^	13.3% (8/60)	12.8% (5/39)	**15.0%** (**9/60)**^a^	5.3% (2/38)	11.7% (7/60)	12.1% (31/257)
	*V. aestuarianus*	**1.7% (1/60)**^a^	0% (0/39)	0% (0/60)	0% (0/38)	**1.7%** (**1/60)**^a^	0.8% (2/257)
Youghal Bay	Haplosporidia spp.	–	9.1% (1/11)	12.5% (2/16)	**56.7%** (**17/30)**^a^	8.33% (1/12)	30.4% (21/69)
	*Minchinia tapetis*	–	0% (0/11)	0% (0/16)	0% (0/30)	0% (0/12)	0% (0/69)
	*Minchinia mercenariae-*like	–	0% (0/11)	**12.5%** (**2/16)**^a^	3.3% (1/30)	0% (0/12)	4.3% (3/69)
	*Vibrio* spp.^b^	–	27.3% (3/11)	**43.8%** (**7/16)**^a^	16.7% (5/30)	33.3% (4/12)	27.5% (19/69)
	*V. aestuarianus*	–	**27.3%** (**3/11)**^a^	6.3% (1/16)	0% (0/30)	0% (0/12)	5.8% (4/69)
Dungarvan Harbour	Haplosporidia spp.	–	**39.2%** (**20/51)**^a^	10.3% (6/58)	30% (9/30)	40% (12/30)	27.8% (47/169)
	*Minchinia tapetis*	–	**2.0%** (**1/51)**^a^	0% (0/58)	0% (0/30)	0% (0/30)	0.6% (1/169)
	*Minchinia mercenariae-*like	–	0% (0/51)	0% (0/58)	0% (0/30)	**10.0%** (**3/30)**^a^	1.8% (3/169)
	*Vibrio* spp.^b^	–	**56.9%** (**29/51)**^a^	**56.9%** (**33/58)**^a^	6.7% (2/30)	36.7% (11/30)	44.4% (75/169)
	*V. aestuarianus*	–	**43.1%** (**22/51)**^a^	0% (0/58)	0% (0/30)	3.3% (1/30)	13.6% (23/169)
Dundalk Bay – Annagassan and Cooley	Haplosporidia spp.	–	**98.3%** (**59/60)**^a^	28.3% (17/60)	45.0% (27/60)	38.3% (23/60)	52.5% (126/240)
	*Minchinia tapetis*	–	**70.0%** (**42/60)**^a^	23.3% (14/60)	0% (0/60)	0% (0/60)	23.3% (56/240)
	*Minchinia mercenariae-*like	–	**11.7%** (**7/60)**^a^	0% (0/60)	1.7% (1/60)	3.3% (2/60)	4.2% (10/240)
	*Vibrio* spp.^b^	–	**38.3%** (**23/60)**^a^	33.3% (20/60)	3.3% (2/60)	26.7% (16/60)	25.4% (61/240)
	*V. aestuarianus*	–	3.3% (2/60)	**10%** (**6/60)**^a^	0% (0/60)	0% (0/60)	3.3% (8/240)
Totals by season	Haplosporidia spp.	45.0% (27/60)	**53.4%** (**86/161)**^a^	25.8% (50/194)	38.6% (61/158)	32.7% (53/162)	37.7% (277/735)
	*Minchinia tapetis*	15.0% (9/60)	**28.6%** (**46/161)**^a^	12.9% (25/194)	0.6% (1/158)	0.6% (1/162)	11.2% (82/735)
	*Minchinia mercenariae-*like	**23.3% (14/60)**^a^	4.3% (7/161)	3.6% (7/194)	1.3% (2/158)	6.8% (11/162)	5.6% (41/735)
	*Vibrio* spp.^b^	13.3% (8/60)	**37.3%** (**60/161)**^a^	35.6% (69/194)	7.0% (11/158)	23.5% (38/162)	25.3% (186/735)
	*V. aestuarianus*	1.7% (1/60)	**16.8%** (**27/161)**^a^	3.6% (7/194)	0.0% (0/158)	1.2% (2/162)	5.0% (37/735)

(–) No samples.

a The highest prevalence (%) for each pathogen group at each site/season is highlighted in bold.

b Potential *Vibrio* species were defined in Table 5.

### Diversity and prevalence of single and multiple infections and the associations between them

**Fig. 2.**
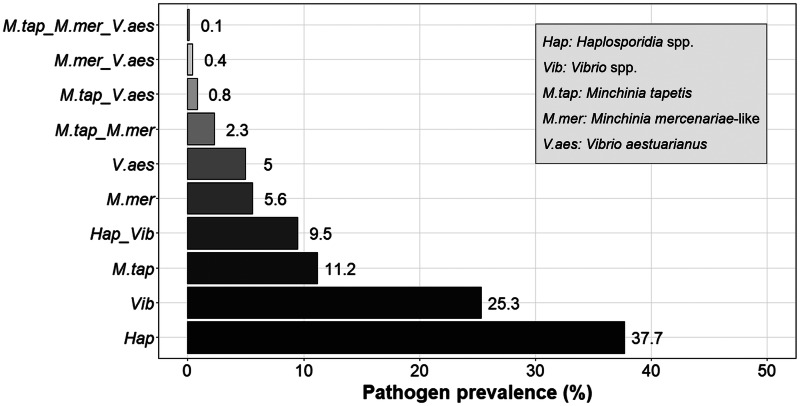
Overall prevalence of infections and coinfections (%) with the different pathogen groups (Hap: Haplosporidia spp.; Vib: *Vibrio* spp.; M.tap: *Minchinia tapetis*; M.mer: *Minchinia mercenariae*-like; V.aes: *Vibrio aestuarianus*).

No OsHV-1 *μ*Var nor microsporidian species were detected in any of the cockles (*n* = 735) screened by PCR. The only pathogen groups detected during the study were Haplosporidia and *Vibrio*. Overall, Haplosporidia was the most common pathogen found through the samples, with a 37.7% (*n* = 277) occurrence (Fig. 2, detailed data in Table 4). Among these 277 infected cockles, two haplosporidian species were identified in the study: *M. tapetis* which was present in 29.6% (*n* = 82/277) of the tested individuals, and *M. mercenariae*-like which was present in 14.8% (*n* = 41/277) (Fig. 2, detailed data in Table 4). Nevertheless, 171 (61.7%) of the tested samples that were positive in the generic haplosporidian PCR did not amplify using the more specific PCR for *Minchinia* spp., which could indicate the presence of other haplosporidian species occurring in the samples. Thus, five of those samples amplified using generic HAP-F1/R3 primers were commercially sequenced; however, low-complexity sequences were obtained, and no significant similarity was found in GenBank. Therefore, the 277 positive PCR cases were defined as Haplosporidia spp. for statistical and analysis purposes.

*Vibrio* was present in 25.3% (*n* = 186) of the total number of cockles screened (Fig. 2, detailed data in Table 4). Of the 186 samples that were positive for *Vibrio*, 19.9% (*n* = 37/186) were confirmed by qPCR to be *V. aestuarianus* (Fig. 2, detailed data in Table 4). *Vibrio aestuarianus*-positive controls (diluted purified DNA) have a *C*_t_ = 30 (60 copies of *V. aestuarianus* DNA per *μ*L). Based on that value, 13.5% (*n* = 5/37) of the individuals were classified as heavily infected individuals (*C*_t_ ⩽ 30), while 86.5% (*n* = 32/37) were classified as low-intensity infections (37 > *C*_t_ > 30).

Direct sequencing was carried out on representative PCR products (*n* = 10) amplified using generic *Vibrio* Vib-F3/R3 primers (Kett *et al*., unpublished as per Notaro *et al*., 2021) to identify other *Vibrio* species present in the samples apart from *V. aestuarianus*. Using BLASTn, three of the sequenced samples had a query length of 127–155 bp with an 83–91% query coverage and 92.6–97.9% identity to *Vibrio splendidus* deposited by Gao (2020) (MT445179.1; MT445177.1) and by Landreau *et al*. (2020) (MT345091.1) (Table 5). Three other PCR product samples that generated sequences had a query length of 82–179 bp, a query coverage of 78–83% and 92.6–98.6% identity to *Vibrio kanaloae* deposited by Zheng (2020) (MT505700.1) and by Barcia and Romalde (2020) (CP065151.1; CP065150.1) (Table 5). Another sample, with a query length of 158 bp, had 94% query coverage and 98% identity to *V. aestuarianus* deposited by Garcia (2018) (MK307696.1) and *Vibrio mediterranei* deposited by Gao *et al*. (2020) (MT269602.1) (Table 5). Two other samples were identified as *Vibrio* spp., as the blasted sequence was quite short (52–69 bp) to differentiate robustly between species (Table 5). The 186 positive PCR cases were defined as *Vibrio* spp. for statistical and analysis purposes.

**Table 5.** Description of the BLASTn results obtained from the sequenced DNA of cockle samples using generic *Vibrio* primers Vib-F3/R3

Sample site	Season	Species identification	Per cent identity	Query cover	Query length (bp)
Youghal Dungarvan Cooley	Summer 2018	*Vibrio spp.* among them: *Vibrio splendidus* and *V. kanaloae*	92.59–98.58%	78–83%	82–179
Annagassan Annagassan	Summer 2018	*Vibrio spp*. most likely *V. splendidus*	97.84–97.92%	88–91%	154–155
Annagassan	Summer 2018	*Vibrio spp.* most likely *V. aestuarianus* or *V. mediterranei*	98.01%	94%	158
Cuskinny Dungarvan	Autumn 2018	*Vibrio spp.*	94.23–94.29%	67–73%	52–69

Overall, 9.5% (*n* = 70/735) of the individuals displayed coinfection with the two pathogen groups present in the samples, Haplosporidia and *Vibrio* (Fig. 2). However, no significant association between them was displayed when testing by SCN (*P* value > 0.05), under the null hypothesis of random pathogen combinations (Table 6), i.e. the pathogen combination was considered not to occur differently than expected by chance.

**Table 6.** Association screening analysis (SCN) results for all the pathogen combinations between Haplosporidia (HAP) and *Vibrio* (VIB) pathogen groups

Coinfection status	Pathogen groups		Obs	LL	UL	SCN *P* value
2-pathogen coinfection	HAP	VIB	70	51	90	0.5174
1-pathogen infection	HAP		207	177	236	0.9832
		VIB	116	92	141	0.5142
Not infected			342	309	376	0.5222

The observed (Obs) frequency of each coinfection status is given along with the lower (LL) and upper (UL) limits of the 95% confidence envelope.

In order to identify the mechanisms driving the pathogen coinfection with Haplosporidia and *Vibrio* pathogen groups, corrected Pearson's χ^2^ test was also performed including seawater temperature, salinity, cockle height and age, as well as the presence/absence of epiflora on the shells (Tables 7 and 8), as confounding factors which may promote pathogen presence. Under the null hypothesis of independence between the two pathogens, the test showed that the observed frequencies for each pathogen combination were not significantly different (Corr *χ*^2^ = 2.51; *ĉ* = 0.84; *P* value 1 = 0.08; *P* value 2 = 0.08) to the expected frequencies considering the confounding factors as drivers of the infection (Supplementary material). Therefore, the proportion of double infected individuals can be explained because the same factors promote the pathogen presence and not because the different pathogen groups are interacting synergistically. The differences in the considered environmental and biotic factors between sample sites and throughout the time, thus, might have been driving the risk of infection in *C. edule* populations.

**Table 7.** Mean ± s.d. of abiotic (seawater temperature and salinity) and biotic factors (cockle height and age) and the overall percentage of the presence of epiflora on the cockle shells at each sample site

Mean ± s.d.	Cork Harbour	Youghal Bay	Dungarvan Harbour	Dundalk Bay
Temperature (°C)	11.8 ± 2.6	10.7 ± 2.9	**12.2 ± 3.0**	10.8 ± 4.2
Salinity (PSU)	33.6 ± 0.8	**33.9 ± 0.03**	**33.9 ± 0.2**	30.4 ± 0.5
Height (mm)	**29.7 ± 7.8**	27.3 ± 4.8	22.0 ± 4.5	29.6 ± 5.3
Age (growth rings)	**3.6 ± 1.9**	2.9 ± 1.7	1.7 ± 1.0	3.0 ± 1.6
Epiflora (presence/absence)	**10.9%**	7.2%	4.7%	1.7%

The largest value for each factor is highlighted in bold.

**Table 8.** Mean ± s.d. of abiotic (seawater temperature and salinity) and biotic factors (cockle height and age) and the overall percentage of the presence of epiflora on the cockle shells by season

Mean ± s.d.	Spring 2018	Summer 2018	Autumn 2018	Winter 2018/19	Spring 2019
Temperature (°C)	12.2 ± 3.9	12.0 ± 3.3	11.9 ± 3.3	11.5 ± 3.4	11.4 ± 3.4
Salinity (PSU)	32.2 ± 1.6	33.1 ± 1.4	32.9 ± 1.6	32.7 ± 1.7	32.6 ± 1.7
Height (mm)	28.0 ± 7.4	27.2 ± 7.1	27.5 ± 6.9	27.8 ± 6.7	27.9 ± 6.6
Age (growth rings)	2.7 ± 1.7	2.8 ± 1.7	2.8 ± 1.8	2.9 ± 1.8	2.9 ± 1.7
Epiflora (presence/absence)	26.7%	8.7%	2.1%	1.9%	4.9%

Regarding the specific species quantified in the samples, only 0.8% (*n* = 6/735) of the individuals were coinfected with *V. aestuarianus* and *M. tapetis*, while 0.4% (*n* = 3/735) were coinfected with *V. aestuarianus* and *M. mercenariae*-like (Fig. 2). Likewise, 13.8% of the *Minchinia*-infected cockles showed a coinfection with both *Minchinia* species (*n* = 17/123); however, the percentage fell to 2.3% when all the screened cockles were considered (*n* = 17/735) (Fig. 2). While coinfection with both *Minchinia* species and *V. aestuarianus* was only observed in one of the samples (0.1%, *n* = 1/735) (Fig. 2).

Associations between *M. tapetis*, *M. mercenariae*-like and *V. aestuarianus* and all the possible combinations were tested by SCN, revealing a significant association between the two species of *Minchinia* (Table 9). The observed number of coinfected individuals with both *Minchinia* species were significantly overrepresented compared to expected by random chance (*P* value < 0.001; Table 9), indicating a significant positive two-way association between these two species. While *M. mercenariae*-like was found to occur alone significantly less often than expected by chance (*P* value < 0.05; Table 9).

**Table 9.** Association screening analysis (SCN) results for all the pathogen combinations between *M. tapetis* (MT), *M. mercenariae-*like (MM) and *V. aestuarianus* (VA)

Coinfection status	Pathogen species			Obs	LL	UL	SCN *P* value
3-pathogen coinfection	MT	MM	VA	1	0	2	0.4292
2-pathogen coinfection	MT	MM		16	0	11	**0**.**0004**
	MT		VA	5	0	10	0.7036
		MM	VA	2	0	6	0.5568
1-pathogen infection	MT			60	53	95	0.0996
		MM		22	21	50	**0**.**0296**
			VA	29	18	46	0.8016
Not infected				600	558	612	0.1836

The observed (Obs) frequency of each coinfection status is given along with the lower (LL) and upper (UL) limits of the 95% confidence envelope. Significant associations (*P* value < 0.05) are highlighted in bold.

The association between *Minchinia* species was further examined by corrected Pearson's *χ*^2^ test in order to assess if the biotic and abiotic factors considered in the study (Tables 7 and 8) influenced this coinfection. The test showed that the observed frequencies for each pathogen combination were significantly different from the expected frequencies considering the confounding factors as drivers of the infection (Supplementary material). The significant outcome (Corr *χ*^2^ = 3.04; *ĉ* = 0.59; *P* value 1 = 0.02; *P* value 2 = 0.02) showed that both pathogens are not independent, and the proportion of double infected individuals cannot be due only to shared confounding factors.

### Site influence on single infections and coinfections

**Fig. 3.**
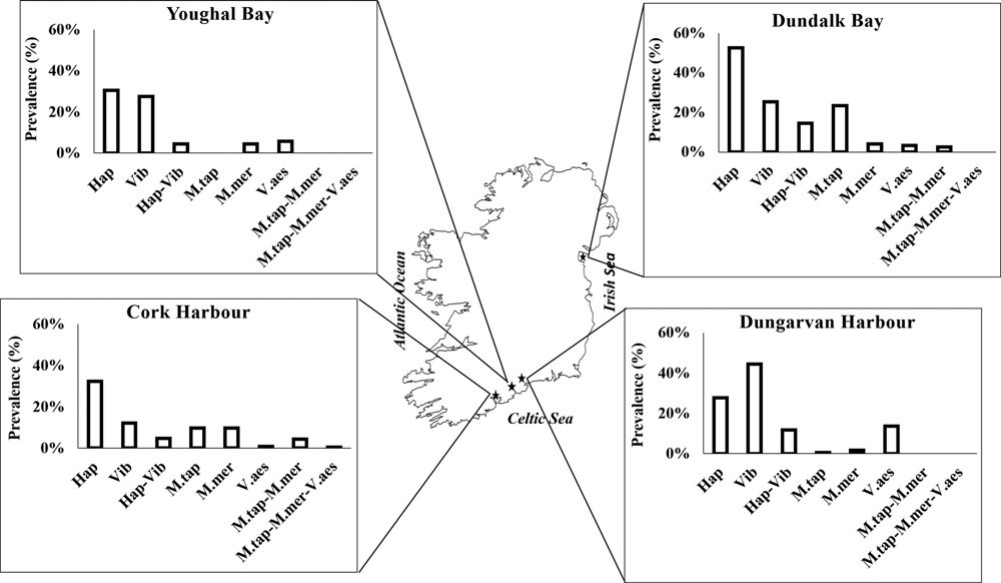
Prevalence (%) of Haplosporidia spp. (Hap), *Vibrio* spp. (Vib), *Minchinia tapetis* (M.tap), *M. mercenariae*-like (M.mer) and *V. aestuarianus* (V.aes) single infections and coinfections (Hap_Vib; M.tap_M.mer; M.tap_M.mer_V.aes) at each sample site.

Spatial variability was assessed and significant differences [*P*(Chisq) < 0.05] were found between the sample sites when the prevalence of single infections and two-pathogen coinfections was considered (Fig. 3). Dundalk Bay was the site with the highest presence of Haploporidia spp. (52.5%, *n* = 126/240), showing a substantially higher prevalence of *M. tapetis* (23.3%, *n* = 56/240) than the other sites, while no presence of *M. tapetis* was observed in Youghal Bay. Dundalk Bay showed lower seawater salinity and a lower presence of epiflora on the cockle shells than the other sample sites (Table 7). Dungarvan Harbour, with the smallest and youngest cockles collected (Table 7), showed the highest presence of *Vibrio* spp. (44.4%, *n* = 75/169) and a peak of *V. aestuarianus* (13.6%, *n* = 23/169). Moreover, three out of the five individuals with the highest intensity of infection (*C*_t_ ⩽ 30) with *V. aestuarianus* were observed at Dungarvan Harbour. Accordingly, the highest prevalence of coinfection with Haplosporidia and *Vibrio* spp. occurred in Dundalk Bay (14.6%, *n* = 35/240) and Dungarvan Harbour (11.8%, *n* = 20/169). However, coinfection with *M. tapetis* and *M. mercenariae*-like was absent in Dungarvan Harbour and Youghal Bay, with Cork Harbour being the site with the highest prevalence (4.3%, *n* = 11/257). The other two-pathogen coinfections with *Minchinia* species and *V. aestuarianus* showed no significant differences between the sites [*P*(Chisq) >0.05] since the total number of positive cases detected was too low [*M. tapetis* and *V. aestuarianus* (*n* = 6); *M. mercenariae* and *V. aestuarianus* (*n* = 3)]. Likewise, Cork Harbour was the only site that showed three-pathogen coinfection with both *Minchinia* species and *V. aestuarianus* (0.4%, *n* = 1/257). Cork Harbour, the site with the oldest cockles, had the highest presence of epiflora on the cockle shells compared to the other sample sites (Table 7).

### Seasonal impact on single infections and coinfections

**Fig. 4.**
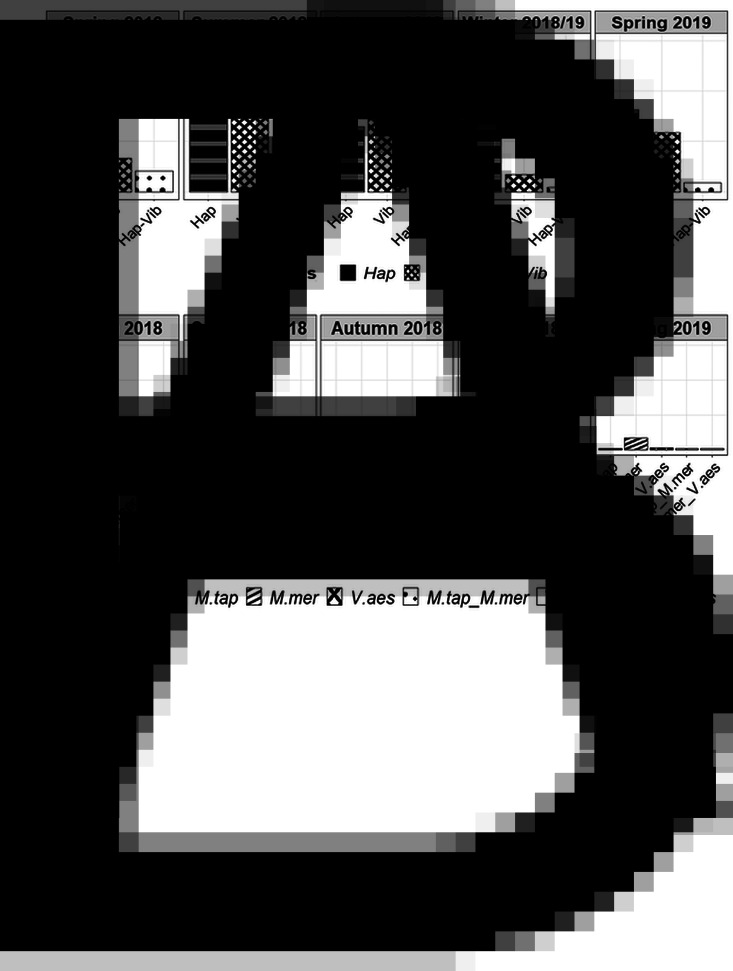
(A) Prevalence (%) of Haplosporidia (Hap) and *Vibrio* (Vib) single infections and coinfection (Hap-Vib) by season. (B) Prevalence (%) of *Minchinia tapetis* (M.tap), *M. mercenariae*-like (M.mer) and *V. aestuarianus* (V.aes) single infections and coinfections (M.tap_M.mer and M.tap_M.mer_V.aes) by season.

Significant seasonal variability [*P*(Chisq) <0.001] was exhibited when the prevalence of single infections and two-pathogen coinfections were compared between seasons (Fig. 4). The absence of *V. aestuarianus* and, in general, a low prevalence of infections were observed in winter 2018/19 (Fig. 4), except for Haplosporidia spp. (38.6%, *n* = 61/158). Summer 2018 had the highest prevalence for all pathogens (Fig. 4), except for *M. mercenariae*-like that exhibited the highest prevalence in spring 2018 (23.3%, *n* = 14/60). Furthermore, the five individuals with the highest intensity of infection (*C*_t_ ⩽ 30) with *V. aestuarianus* were observed during summer 2018. The highest prevalence of coinfection with Haplosporidia and *Vibrio* spp. occurred in summer 2018 (21.7%, *n* = 35/161), while the highest coinfection prevalence with *M. tapetis* and *M. mercenariae*-like occurred in spring 2018 (11.7%, *n* = 7/60), with no occurrence in winter 2018/19. The higher pathogen incidence during spring and summer corresponded with higher seawater temperature and a peak in the presence of epiflora on the cockle shells (Table 8). The other two-pathogen coinfections with *Minchinia* species and *V. aestuarianus* showed no significant differences throughout the year [*P*(Chisq) > 0.05] since the total number of positive cases detected was too low [*M. tapetis* and *V. aestuarianus* (*n* = 6); *M. mercenariae* and *V. aestuarianus* (*n* = 3)]. There was only one case of three-pathogen coinfection with *M. tapetis*, *M. mercenariae*-like and *V. aestuarianus* in spring 2019 (0.6%, *n* = 1/162).

### Histopathology

**Fig. 5.**
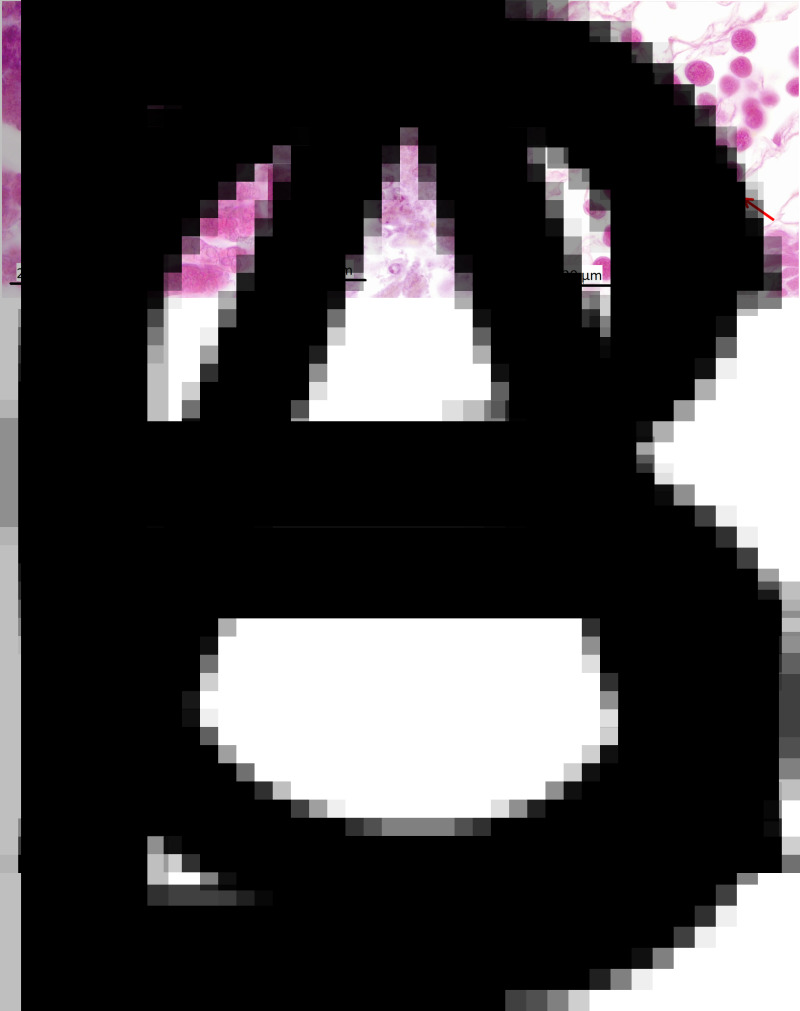
(A) Multiple haplosporidia-like sporonts (black arrow) along with haemocytes (red arrow) in the connective tissue of *Cerastoderma edule*; (B) a large number of haplosporidian spores (black arrow) in the mantle tissue of *C. edule*; and (C) red arrow shows a haemocyte accumulation in the connective tissue of *C. edule*.

The histological screening was used as a validation method of the PCR and qPCR results. Haplosporidian infection was observed in histological sections of 67 cockles out of a subsample of 70 samples (95.7% visual confirmation) that were positive for generic haplosporidian PCR. Mostly haplosporidians were observed as multinucleate plasmodia, multiple haplosporidia-like sporonts and developing spores similar to those described for *Minchinia* spp. (Ramilo *et al*., 2018; Lynch *et al.*, 2020*b*; Fig. 5), and as uninucleate cells stage in lower numbers. Among the sample sites, the haplosporidia-like sporonts were predominantly visualized in the infected individuals from Cork Harbour in spring 2018, autumn 2018 and spring 2019; while the developing spores were observed mainly in the infected individuals from Dundalk Bay in summer 2018 and winter 2018/19. A higher prevalence of haplosporidians was recorded by PCR at both sites. The multinucleate plasmodia were visualized in cockles throughout the year at the different sample sites. The multinucleate plasmodia and haplosporidia-like sporonts were mainly observed throughout the connective tissue of the gills and digestive gland, while the developing spores were principally found in the mantle of infected animals.

Host pathology associated with *Vibrio* species included haemocyte accumulation in the connective tissues, as McCleary and Henshilwood (2015) described in *C. gigas* infected with *V. aestuarianus*. In this study, some of the sections examined (*n* = 28) showed haemocyte accumulation in the connective tissues of *C. edule* (Fig. 5). However, the presence of haemocyte infiltrations throughout the organs and tissues has also been previously associated with *Minchinia* infection (Longshaw and Malham, 2013). In this study, only in a minor number of screened samples (*n* = 4), where *Vibrio* and Haplosporidia were detected by PCR, haemocyte infiltrations were observed, all of them at Cork Harbour.

Although OsHV-1 *μ*Var is not visible using histology, cytopathic effects can be observed in infected hosts, such as hypertrophied cells with a low nucleus-cytoplasm ratio and nuclear abnormalities (Prado-Alvarez *et al*., 2016). Abnormal cell pathology associated with a viral infection was not observed in the histology (*n* = 70). Similarly, microsporidial spores may be visualized by microscopy in infected hosts (Garcia, 2002); however, it was not observed in the histological sections screened (*n* = 70).

Ten histological sections of samples that were negative by PCR were screened and no evident signal of infection was found in those, and tissues, organs and sinuses presented good integrity and a clear structure.

## Discussion

The present study describes for the first time, to the best of our knowledge, simultaneous infections with haplosporidians and *Vibrio* spp. in wild and fished *C. edule* populations along the Irish coast. Coinfections with two and, especially, three pathogens, were rare in this study compared to single infected individuals. This finding may indicate that coinfected individuals succumb more easily to simultaneous infections and die. The lower number of coinfected individuals screened might indicate that the sampling was carried out on a much smaller cohort of survivors within the population. This survival bias is supported by previous studies on mortalities associated with coinfections of ostreid herpesvirus (OsHV-1 and variants) and *Vibrio* spp. in *C. gigas* in France (Petton *et al*., 2015; Azéma *et al*., 2016; Petton *et al*., 2019), as well as with the co-occurrence of different protozoa and bacteria in the noble pen shell *Pinna nobilis* in the Mediterranean Sea (Carella *et al*., 2020). Only three individuals presented a three-pathogen coinfection with *Minchinia* spp. and *Vibrio* spp., all located at Cork Harbour. Cork Harbour was the site with the oldest cockles, and it is well known that older hosts have more time than younger hosts to accumulate disease-causing pathogens (Lafferty and Kuris, 2009; Breitburg *et al*., 2015). Nevertheless, older bivalves have also more time available to develop immunotolerance and defence mechanisms compared to their younger cohorts (Renault and Novoa, 2004; Coen and Bishop, 2015). It may be the reason why cockles at Cork Harbour have not succumbed to the multiple infections.

Even though Haplosporidia and *Vibrio* were found simultaneously in double infected individuals, no significant biological interaction was statistically identified between those pathogen groups. Unlike other studies that have reported that multiple pathogens can interact synergistically, with the presence of one pathogen enhancing the abundance and/or virulence of the other (De Lorgeril *et al*., 2018; Carella *et al*., 2020). Concurrent infections by multiple pathogens species are more likely to occur in immune depressed individuals, which are more vulnerable to multiple opportunistic infections (De Lorgeril *et al*., 2018; Carella *et al*., 2020). These findings highlight that there can be variation amongst pathogen groups and the primary pathogen–opportunistic pathogen interplay.

It is well known that environmental and ecological factors potentially influence the mechanisms of disease transmission in marine systems, i.e. hydrodynamics, the biomass of infected animals, etc. (Petton *et al*., 2015). Similarities in the host environment, behaviour or susceptibility can cause correlations in the risk of infection between two parasites (Vaumourin *et al*., 2014). In this study, the proportion of double infected individuals with Haplosporidia and *Vibrio* was explained by risk factors (increased seawater temperature, reduced salinity and host condition) shared by the two pathogen groups, increasing the probability of infection by both infectious agents. Such confounding factors may influence host exposure and/or host susceptibility promoting simultaneous infections, even though involved pathogens do not interact biologically (Vaumourin *et al*., 2015). These factors may be naturally occurring or be outcomes of coastal development (e.g. pollution) or climate change (e.g. warming, increased precipitation events and subsequent freshwater loadings into bays and estuaries), and may impact the expression of diseases directly or indirectly (Coen and Bishop, 2015). The predicted warming, increased precipitation and reduced salinity of coastal waters in temperate regions will provide new areas for the natural occurrence of pathogenic strains and may also impact the resilience of bivalve hosts (Rowley *et al*., 2014; Lynch *et al*., 2020*a*).

Consequently, spatial variability in the pathogen infection was detected driven by environmental and biological variables. The highest prevalence of coinfection with Haplosporidia and *Vibrio* spp. occurred in Dundalk Bay, which had a lower mean salinity throughout the year compared to the other sites. Gorrasi *et al*. (2020) observed that the abundance of *Vibrio* genus in water decreased along the salinity gradient within a marine saltern hypersaline environment. In general, marine vibrios are considered halotolerant, with no extreme halophiles reported within this genus (Gorrasi *et al*., 2020). Previous studies have highlighted the intolerance of haplosporidians to low salinity in *C. edule* (Albuixech-Martí *et al*., 2020), eastern oyster *Crassostrea virginica* (Ford and Haskin, 1982; Burreson and Ford, 2004) and European flat oyster *Ostrea edulis* (Flannery *et al*., 2014). The high prevalence of haplosporidians at Dundalk Bay may be due to its commercial fishery activity. It is recognized that fishing and dredging may unbalance the interplay between infectious disease agents and their hosts in marine ecosystems (Rowley *et al*., 2014; Coen and Bishop, 2015). Cranfield *et al*. (1999, 2005) hypothesized that extensive dredging in Foveaux Strait of New Zealand contributed to increased densities of Chilean oysters (*Ostrea chilensis*) and decreased densities of other filter-feeding organisms, that may have previously reduced the dispersal stages of the haplosporidian *Bonamia exitiosa*, enhancing disease transmission. The high cockle density found at Dundalk Bay may have facilitated the infection among the cockle population, due to the proximity of infected to non-infected cockles. The cultivated and harvested molluscs tend to have a much greater incidence of disease than wild molluscs, which may be because they are placed at high densities which enhance disease transmission and cause stress (competition for resources, space, etc.) among individuals (Ford, 2002).

Dungarvan Harbour also displayed a high prevalence of coinfection with Haplosporidia and *Vibrio* spp. compared to the other sites. Cockles from Dungarvan Harbour were the smallest and the youngest ones. Although it is accepted that older hosts have more time to accumulate disease-causing pathogens (Lafferty and Kuris, 2009; Breitburg *et al*., 2015), the susceptibility of small bivalves to viral and bacterial infections is generally greater than for larger adults (Renault and Novoa, 2004; Coen and Bishop, 2015). Moreover, the proximity of an extensive *C. gigas* culture site at Dungarvan Harbour, where *Vibrio* spp. are endemic (pers. comm. with Marine Institute Ireland), can have promoted the presence of *Vibrio* in the environment. Particularly, *V. aestuarianus* and *V. splendidus*, which have been identified in this study, have been described as increasingly harmful pathogens of *C. gigas* aquaculture (Le Roux *et al*., 2002; Garnier *et al*., 2007; McCleary and Henshilwood, 2015). Caballes *et al*. (2012) demonstrated interspecific transmission of *Vibrio rotiferianus* between co-occurring echinoderm species.

Even though the natural host for OsHV-1 are *C. gigas*, this virus can behave opportunistically and infect other cohabiting bivalve species (Bookelaar *et al*., 2020). It has been previously reported in *C. edule*, in Ireland (Bookelaar *et al*., 2020) and in the Sydney cockle, *Anadara trapezia*, in Australia (Evans *et al*., 2017). Nevertheless, no presence of OsHV-1 *μ*Var was detected in the study, despite the extensive farming of *C. gigas* at Dungarvan Harbour, which has a history of OsHV-1-associated mortality events (Lynch *et al*., 2012; Prado-Alvarez *et al*., 2016). During summer 2018, the prevalence of OsHV-1 *μ*Var in *C. gigas* at Dungarvan Harbour was confirmed by Kett *et al*. (unpublished data) to be 48.4% (*n* = 92/190), with low mortality rates (max. of 6% at the high shore in July). Moreover, Bookelaar *et al*. (2020) observed OsHV-1*μ*Var-infected *C. edule* (14.4%; *n* = 36/250) and infected *C. gigas* (range of 0–27% per month; *n* = 270) at Dungarvan Harbour from April 2015 to August 2015, with no recording of mortalities. Thus, the lack of OsHV-1 *μ*Var detection in *C. edule* at Dungarvan Harbour in the current study may be because the virus remained within the oysters and was not shed from dying or dead oysters into the environment, as the high prevalence and low mortality recorded by Kett *et al*. (unpublished data) suggested. The smaller sample size (*n* = 81), compared to the above studies, taken during spring and summer 2018, when the OsHV-1 *μ*Var can have a major emergence due to the higher temperatures (Renault *et al*., 2014), may have also influenced the results.

Seasonal variability was also observed in this study, with the highest prevalence of coinfection with Haplosporidia and *Vibrio* spp. occurring in summer 2018, which coincided with the highest intensity of infection with *V. aestuarianus.* Additionally, lower occurrences of the haplosporidian and *Vibrio* species quantified were detected in the cooler spring 2019 compared to spring 2018 in this study. Burreson and Ford (2004) tested that low-temperature conditions caused a dramatic reduction in the prevalence of *Haplosporidium nelsoni* in *C. virginica*. Conversely, a higher prevalence of *Bonamia ostreae* in European flat oyster *O. edulis* was associated with cooler spring temperatures over a 30-year study period in Ireland (Engelsma *et al*., 2010; Lynch *et al*., 2014). These findings would suggest that a thermal tolerance range exists for haplosporidian species. It is also well-known that bacteria belonging to the genus *Vibrio* are strongly thermo-dependent and their occurrence is expected to rise with increasing temperature conditions (Garnier *et al*., 2007; Cotter *et al*., 2010; Vezzulli *et al*., 2010). Most of the reported *Vibrio*-associated mass mortalities in marine invertebrates have occurred during the summer when seawater temperatures were higher than 18°C (Le Roux *et al*., 2002; Paillard *et al*., 2004; Garnier *et al*., 2007; Vezzulli *et al*., 2010). Therefore, high temperatures in summer might have triggered a major pathogen propagation and, consequently, to a higher prevalence of two-pathogen coinfection. While lower temperatures in winter, with the lowest prevalence of two-pathogen coinfection, would have kept the pathogens at a sub-lethal level in the hosts reducing the transmission between cockles. It has been observed that *Vibrio* spp. may enter into a dormant state when the environmental conditions become unfavourable (i.e. suboptimal or reduced temperature, elevated salinity, low nutrient concentration and extreme pH) (Lin and Schwarz, 2003; Nowakowska and Oliver, 2013; Neogi *et al*., 2018). The low number of coinfected individuals in winter might also suggest that most *C. edule* had succumbed to infection in autumn after the peak in summer.

It was also observed that with the higher temperatures in summer and spring, the presence of epiflora on the cockle shells was more frequent, which may have consequences on cockle susceptibility to infectious agents. As seen in shellfish aquaculture, the presence of biofouling on the stock can have a direct impact, causing physical damage, biological competition, mechanical interference and environmental modification, while infrastructure is also impacted (Fitridge *et al*., 2012). Microalgae have been pointed as a repetitive and substantial source of bacterial inoculation into the bivalve larval culture systems (Salvesen *et al*., 2000). Microalgae may promote and/or inhibit bacterial growth by the production of organic exudates and toxic metabolites (Salvesen *et al*., 2000). For instance, *Vibrio* species such as *V. splendidus* or *V. parahaemolyticus* have been found to be associated with microalgae (Kumazawa *et al*., 1991; Rehnstam-Holm *et al*., 2010; Notaro *et al*., 2021). Therefore, the presence of epiflora on the shells might have also exposed the cockles to pathogens, in particular *Vibrio* species.

Both species of *Minchinia*, *M. tapetis* and *M. mercenariae*-like, were found to occur together significantly more often than expected by random chance, indicating a positive two-way association between both species. However, previous studies examining pairwise coinfections indicate that antagonistic interactions between similar types of parasites may be common (Dobson, 1985; Johnson and Hoverman, 2012). A possible explanation of the established synergetic interaction is that, despite both *Minchinia* species being closely related, they exhibit differences in their pathogenic effect and use of resources within the host. Carballal *et al*. (2020) observed that the distribution of infection with *M. tapetis* and *M. mercenariae*-like haplosporidian in cockles *C. edule* in Galicia was different throughout the cockle tissues. *M*. *mercenariae*-like haplosporidian was observed in the connective tissue of the digestive gland, gills and gonad, while *M. tapetis* was only observed in the digestive gland (Carballal *et al*., 2020). Further research is needed to reveal the underlying mechanism of this synergetic association in *C. edule* and the role that host exposure and host habitat play in it.

In brief, coinfected individuals may more easily succumb to pathogen load. The proportion of double infected individuals with haplosporidian and *Vibrio* was driven by environmental and biological factors, triggering a spatial and seasonal variability in the distribution of coinfection. The higher temperature in warmer months and the presence of epiflora, which can be associated with pathogens, on the cockle shells may have promoted the risk of coinfection, along with low salinity and the host condition in some of the sample sites. The close proximity to other infected host species may have also influenced the coinfection distribution in *C. edule*. Similarly, fished *C. edule* populations with high cockle densities might have been more exposed to the infection. Therefore, those factors may conform the ideal scenario for simultaneous infections, although the pathogen groups showed no biological interaction between them. Nevertheless, a positive two-way association between both *Minchinia* species was observed, which is suggested to be due to their different pathogenic effect and use of resources within the host. Our findings shed light on the complex interactions between multiple aetiological agents associated with host diseases in the frame of a natural system. This study highlights the consequences of the coinfections are not simply the sum of the effects caused by each infectious agent, but the result of a complex combination of direct and indirect pathogens effects, the host ecology and the environmental context.

These results are framed into the current scenario of climate change, which involves oceans warming on average by 0.6°C in the next 100 years (Wiltshire *et al*., 2008), with European seas especially affected (Philippart *et al*., 2011). The sea surface temperature around the UK and Ireland has increased at six times the rate of the global average, particularly jeopardizing species that have small thermal tolerance windows (Frost *et al*., 2012). As a result, previously appropriate habitats for cockles may no longer be suitable due to changes in thermal regime, oxygen levels or altered rainfall patterns (Morgan *et al*., 2013; Coen and Bishop, 2015). Climate change may be also expected to have a large effect on disease occurrence, especially in host species showing strong relationships between temperature/salinity and diseases, as seen for the cockle populations studied, and for many other molluscan host–parasite systems (Allam and Raftos, 2015; Coen and Bishop, 2015; Lynch *et al*., 2020*a*). The physiological stress may also cause the host to become immune-compromised and unable to suppress an infection (Suttle, 2007). Therefore, climate change could be the driver of the spreading and range expansions of infectious agents, such as bacteria, haplosporidians and viruses, in the marine ecosystem and may lead to more frequent pathogen outbreaks (Burreson and Ford, 2004; Suttle, 2007; Rowley *et al*., 2014; Coen and Bishop, 2015). As global climate change and anthropogenic impacts continue to modify the distribution and abundance of hosts and parasites and the ways in which they interact, integral studies like ours are important since concepts gained might be applied to other associations and to a changing environment.

A final important point to emphasize is that the relative potential of coinfections to cause the emergence of zoonoses in wild animals remains to be fully assessed (Hoarau *et al*., 2020). Given that two-thirds of emerging infectious diseases are zoonoses, with nearly 70% originating from wildlife (Hoarau *et al*., 2020), better knowledge of the interactions of infectious agents in wild reservoirs, such as cockles, will provide key insight for the understanding and management of spillover processes. Although challenging, findings from this study highlight the necessity to integrate data pertaining to host ecology, pathogen diversity, biogeography and seasonality of the disease and the environmental influence. This approach should be consistently applied to provide a more detailed picture of coinfection dynamics in individual hosts and communities over time.

## Supplementary material

For supplementary material accompanying this paper visit https://doi.org/10.1017/S0031182021001396. click here to view supplementary material
