# Supplementary material for: Co-occurrence of pathogen assemblages in a keystone species the common cockle Cerastoderma edule on the Irish coast
Source: Parasitology. 2021 Jul 30;148(13):1665–79. doi: 10.1017/S0031182021001396 (PMC8564771; doi:10.1017/S0031182021001396)
Supplement: Supplementary file 1 [file S0031182021001396sup.zip › S0031182021001396sup002.docx]

**Supplementary material**

Suppl. Table 1: Observed frequencies of infection with Haplosporidia and *Vibrio* and their combination; and expected frequencies under corrected Pearson’s chi-square test considering confounding factors.

| Corrected Pearson’s Chi-square Test | | |
| --- | --- | --- |
| *Haplosporidia-Vibrio* | **Observed frequencies:** | **Expected frequencies:** |
| -/- | 342 | 351.17 |
| -/+ | 116 | 106.83 |
| +/- | 207 | 197.83 |
| +/+ | 70 | 79.17 |

(-/-) Non-infected individuals

(-/+) (+/-) Single infected individuals

(+/+) Double infected individuals

Suppl. Table 2: Observed frequencies of infection with *Minchinia tapetis* and *M. mercenariae*-like and their combination; and expected frequencies under corrected Pearson’s chi-square test considering confounding factors.

| Corrected Pearson’s Chi-square Test | | |
| --- | --- | --- |
| *M. tapetis* and  *M. mercenariae-like* | **Observed frequencies:** | **Expected frequencies:** |
| -/- | 171 | 166.28 |
| -/+ | 24 | 28.72 |
| +/- | 65 | 69.72 |
| +/+ | 17 | 12.28 |

(-/-) Non-infected individuals

(-/+) (+/-) Single infected individuals

(+/+) Double infected individuals
