# Supplementary figures and images for: Co-occurrence of pathogen assemblages in a keystone species the common cockle Cerastoderma edule on the Irish coast
Source: Parasitology. 2021 Jul 30;148(13):1665–79. doi: 10.1017/S0031182021001396 (PMC8564771; doi:10.1017/S0031182021001396)

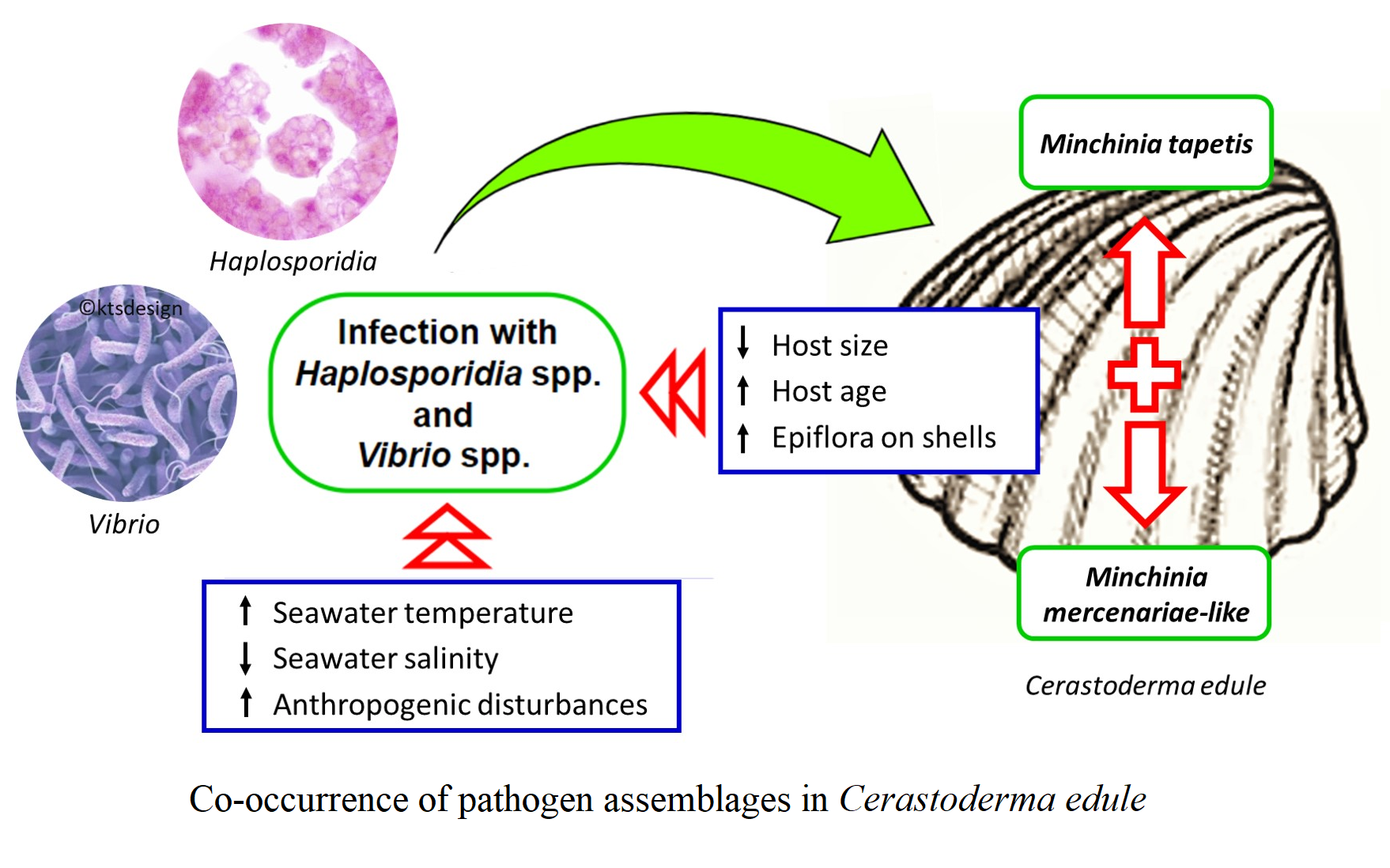

Supplement: Supplementary file 1 [file S0031182021001396sup.zip › S0031182021001396sup001.tif]
